# Genetic diversity among *Trypanosoma (Duttonella) vivax* strains from Zambia and Ghana, based on cathepsin L-like gene

**DOI:** 10.1051/parasite/2013024

**Published:** 2013-07-01

**Authors:** Jesca Nakayima, Ryo Nakao, Andy Alhassan, Kyoko Hayashida, Boniface Namangala, Charles Mahama, Kofi Afakye, Chihiro Sugimoto

**Affiliations:** 1 Division of Collaboration and Education, Research Center for Zoonosis Control, Hokkaido University Kita 20, Nishi 10 Kita-ku, Sapporo Hokkaido 001-0020 Japan; 2 National Livestock Resources Research Institute (NaLIRRI) P.O. Box 96 Tororo Uganda; 3 Veterinary Services Department P.O. Box M161 Accra Ghana; 4 Department of Paraclinical Studies, School of Veterinary Medicine, University of Zambia P.O. Box 32379 Lusaka Zambia

**Keywords:** Trypanosomiasis, *Trypanosoma vivax*, Zambia, Ghana

## Abstract

Understanding the evolutionary relationships of *Trypanosoma (Duttonella) vivax* genotypes between West Africa and Southern Africa can provide information on the epidemiology and control of trypanosomosis. Cattle blood samples from Zambia and Ghana were screened for *T. vivax* infection using specie-specific PCR and sequencing analysis. Substantial polymorphism was obtained from phylogenetic analysis of sequences of cathepsin L-like catalytic domains. *T. vivax* from Ghana clustered together with West African and South American sequences, while *T. vivax* from Zambia formed one distinct clade and clustered with East African and Southern African sequences. This study suggests existence of distinct genetic diversity between *T. vivax* genotypes from West Africa and Zambia as per their geographical origins.

## Abstract (fr)

Comprendre les relations évolutives des génotypes de *Trypanosoma (Duttonella) vivax* d’Afrique de l’Ouest et du Sud peut fournir des informations sur l’épidémiologie et le contrôle de la trypanosomiase. Des échantillons de sang de bétail de Zambie et du Ghana ont été testés pour l’infection à *T. vivax* en utilisant une PCR spécifique à l’espèce et en analysant les séquences. Un polymorphisme substantiel a été obtenu par une analyse phylogénétique des séquences de domaines catalytiques similaires à la cathepsine L. *T. vivax* du Ghana était groupé avec les séquences d’Afrique de l’Ouest et d’Amérique du Sud, alors que *T. vivax* de Zambie formait un clade distinct et était groupé avec des séquences d’Afrique de l’Est et du Sud. Cette étude suggère l’existence d’une diversité génétique distincte des génotypes de *T. vivax* d’Afrique de l’Ouest et de Zambie en fonction de leurs origines géographiques.

Food and Agriculture Organization (FAO) has estimated that the problem of trypanosomosis costs Africa about US$4.5 billion per year, which includes losses in agricultural production, perennial expenditure on trypanocidal drugs, and other local intervention schemes in attempts to control trypanosomosis [13, 19]. Of major importance is *T. vivax* infection that predominantly affects cattle, buffalo, goats, sheep, and wild bovids [7, 18]. *T. vivax* is a hemoprotozoan parasite found in Africa, central and South America. In Africa the parasite is transmitted both cyclically by *Glossina* spp. and mechanically by tabanids and other biting flies, while in South America only mechanical transmission occurs [3, 22, 26, 29]. Certain cattle and goat breeds in Africa are trypanotolerant while others are highly susceptible to *T. vivax* infection.

*T. vivax* infection in small ruminants from Africa and South America has been reported to result in a variety of clinical outcomes ranging from acute to chronic or subclinical disease; the course of infection varying depending on the parasite strain, endemicity, and the species and breed of the ruminant host [2, 4, 5, 10]. West African *T. vivax* strains are believed to be more pathogenic compared to East African strains, however, severe hemorrhagic outbreaks, with high mortality levels, have been periodically reported in East Africa [6, 21]. South American *T. vivax* strains have been shown to have close genetic similarity to West African strains [7, 14], re-affirming the historical theory that *T. vivax* was imported into the New World through infected cattle from West Africa [18]. Although *T. vivax* is considered to be an important salivarian trypanosome species because of its wide distribution, pathogenicity to cattle, and its relatively high infection rates in tsetse, it remains highly neglected in the scientific literature. This is attributed to the species being notoriously difficult to work with as isolates are not easily adapted to culture or grown in standard laboratory animals [15]. Few studies that have investigated the genetic diversity in *T. vivax* have typically focused on comparison between isolates from across Africa and South America [1, 11].

The genomes of trypanosomes consist of multiple copies of catL-like genes that vary depending on species. Cathepsin L-like (catL-like) enzymes are cysteine proteases involved in the pathogenicity, immunity, cell differentiation, infectivity, and metabolism of trypanosomes [8]. The potential role of cysteine proteases in the life cycle and pathogenesis of trypanosomes can be elucidated by knowledge of their evolutionary relationships and identification of species-specific molecules [20, 25]. In this study, we characterized sequences of cathepsin L-like genes of *T. vivax* circulating in Ghana (West Africa) and Zambia (Southern Africa) and compared their relationship by phylogenetic analysis.

A total of 100 bovine blood samples from Petauke district in Zambia (Latitude: −14° 14′ 28.5246′′ and Longitude: 31° 19′ 11.4564′′) were screened for *T. vivax* infection using the species-specific PCR (designated TviCatL-PCR) employing primers TviCatL1 and DTO155 [8]. Thereafter, *T. vivax*-positive samples (*n* = 11) were randomly selected and subjected to PCR amplifying approximately 500 bp of cathepsin L-like gene using primers DTO154 and DTO155 [8]. The bovine blood samples from Adidome and Koforidua in Ghana, which were positive for *T. vivax* in a previous study [24], were also included in this study (*n* = 11). PCR was conducted using Amplitaq Gold^®^ 360 reagent (Applied Biosystems, Foster City, CA) with 35 cycles of amplification to minimize PCR errors. The amplified products were cloned into a pGEM-T vector (Promega, Madison, WI) and sequenced using the BigDye Terminator version 3.1 Cycle Sequencing Kit (Applied Biosystems) and an ABI Prism 3130× genetic analyzer according to the manufacturers’ instructions. The DNA sequences obtained were submitted to the DNA Data Bank of Japan (DDBJ) (http://www.ddbj.nig.ac.jp) under Accession Nos. AB781071 to AB781078.

Among 100 cattle tested, 32 samples were positive for *T. vivax* infection (data not shown). The infection rate (32%) was comparable to that observed in Ghanaian cattle (25.3%) in our previous study [24]. Seven different sequences were obtained from 11 Zambian samples, while all the 11 samples from Ghana had an identical sequence. A phylogenetic analysis revealed that *T. vivax* from Ghana clustered together with those from West Africa (Burkina Faso) and South America (Brazil and Venezuela) (Figure 1). *T. vivax* from Zambia were assigned to three different clades, forming one distinct clade.

**Figure 1.**
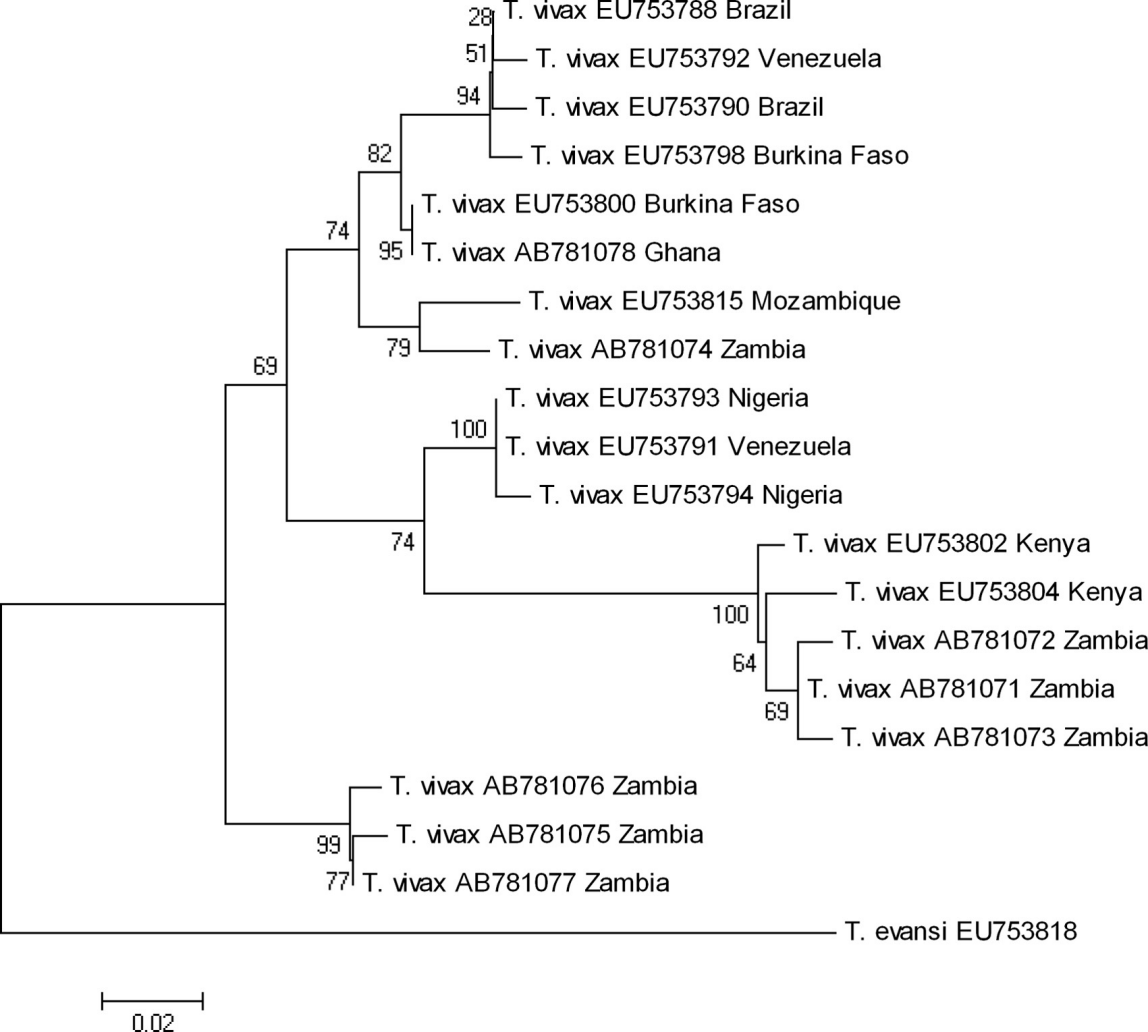
Phylogeny based on Neighbor-joining method using catL-like catalytic domain sequences from West African (Ghana) and Southern African (Zambia) isolates of *T. vivax*. The numbers correspond to percentage of bootstrap support values derived from 1000 replicates. Comparison with GenBank sequences with their accession nos. for South American, Southern African, East African, and West African *T. vivax* is indicated.

Genetic diversity of *T. vivax* has been reported to be limited [27]. It is, however, essential to know its true diversity considering the differences in disease outcome, diagnosis, response to drug, and resistance to chemotherapeutic treatments [17]. Clustering of Ghanaian *T. vivax* with those from West Africa and South America is in agreement with previous findings [11, 12, 14], corroborating the hypothesis that *T. vivax* was introduced into South America with bovines imported from West Africa [11, 12, 16, 18]. *T. vivax* from Central Africa is reported to share molecular features with both the East and West African isolates [14, 15]. Previously, other workers [16, 7, 23, 28, 14, 11] illustrated a complex structure of *T. vivax* populations corroborating the high genetic divergence between West and East African *T. vivax* strains. This has been revealed according to morphological, immunological, pathological, molecular features, and behavior in the tsetse fly and mammalian hosts.

An attempt to characterize *T. vivax* using a panel of eight microsatellites [9] failed to amplify any positive alleles from our *T. vivax*-positive samples (data not shown). Such failure has been attributed to a population whose parasitemia is below the threshold for detection by single-locus PCR [9]. Additionally, there exist high levels of genetic diversity within this species, particularly in East Africa, and primer sets used for microsatellite genotyping were designed from genomic sequences from a West African strain. The diverse genotypes in East Africa and the need to identify novel genotypes require use of “generic” primers, designed to amplify DNA from a wide range of trypanosome species [17]. In conclusion, we revealed the genetic diversity of *T. vivax* genotypes from Ghana (West Africa) and Zambia (Southern Africa).

## References

[1] Allsopp BA, Newton SD. 1985 Characterization of *Trypanosoma (Duttonella) vivax* by isoenzyme analysis. International Journal for Parasitology, 15, 265–270 4030202 10.1016/0020-7519(85)90063-3

[2] Anosa VO. 1983 Diseases produced by *Trypanosoma vivax* in ruminants, horses and rodents. Veterinary Medicine, 30, 717–741 10.1111/j.1439-0450.1983.tb01898.x 6367315

[3] Applewhaite LM. 1990 Small ruminant trypanosomiasis in Guyana–a preliminary report. British Veterinary Journal, 146, 93–94 2306609 10.1016/0007-1935(90)90083-F

[4] Batista JS, Bezerra FS, Lira RA, Carvalho JRG, Neto AMR, Petri AA, Teixeira MMG. 2008 Aspectos clinicos, epidemiologicos e patologicos da infecção natural em bovinos por *Trypanosoma vivax* na Paraiba. Pesquisa Veterinária Brasileira, 28, 63–69

[5] Batista JS, Oliveira AF, Rodrigues CMF, Damasceno CAR, Oliveira IRS, Alves HM, Paiva ES, Brito PD, Medeiros JMF, Rodrigues AC, Texeira MMG. 2009 Infection by *Trypanosoma vivax* in goats and sheep in the Brazilian semiarid region: from acute disease outbreak to chronic cryptic infection. Veterinary Parasitology, 165, 131–135 19665308 10.1016/j.vetpar.2009.07.005

[6] Catley A, Irungu P, Simiyu K, Dadye J, Mwakio W, Kiragu J, Nyamwaro SO. 2002 Participatory investigations of bovine trypanosomiasis in Tana River District, Kenya. Medical and Veterinary Entomology, 16, 55–66 11963982 10.1046/j.0269-283x.2002.00346.x

[7] Cortez AP, Ventura RM, Rodrigues AC, Batista JS, Paiva F, Añez N, Machado RZ, Gibson WC, Teixeira MMG. 2006 The taxonomic and phylogenetic relationships of *Trypanosoma vivax* from South America and Africa. Parasitology, 133, 159–169 16650339 10.1017/S0031182006000254

[8] Cortez AP, Rodrigues AC, Garcia HA, Neves L, Batista JS, Bengaly Z, Paiva F, Teixeira MMG. 2009 Cathepsin L-like genes of *Trypanosoma vivax* from Africa and South America – characterization, relationships and diagnostic implications. Molecular and Cellular Probes, 23, 44–51 19063960 10.1016/j.mcp.2008.11.003

[9] Duffy CW, Morrison LJ, Black A, Pinchbeck GL, Christley RM, Schoenefeld A, Tait A, Turner CMR, MacLeod A. 2009 *Trypanosoma vivax* displays a clonal population structure. International Journal for Parasitology, 39, 1475–1483 19520081 10.1016/j.ijpara.2009.05.012

[10] Desquesnes M. 2004 Livestock Trypanosomoses and their Vectors in Latin America. Paris: OIE & CIRAD, 190 pp.

[11] Dirie MF, Murphy NB, Gardiner PR. 1993a DNA fingerprinting of *Trypanosoma vivax* isolates rapidly identifies intraspecific relationships. Journal of Eukaryotic Microbiology, 40, 132–134 8461886 10.1111/j.1550-7408.1993.tb04892.x

[12] Dirie MF, Otte MJ, Thatthi R, Gardner PR. 1993b Comparative studies of *Trypanosoma (Duttonella) vivax* isolates from Colombia. Parasitology, 106, 21–29 8097584 10.1017/s0031182000074771

[13] Eisler MC, Torr SJ, Coleman PG, Machila N, Morton JF. 2003 Integrated control of vector-borne diseases of livestock – pyrethroids: panacea or poison? Trends in Parasitology, 19, 341–345 12901934 10.1016/s1471-4922(03)00164-8

[14] Fasogbon AI, Knowles G, Gardiner PR. 1990 A comparison of the isoenzymes of *Trypanosoma (Duttonella) vivax* isolates from East and West Africa. International Journal for Parasitology, 20, 389–394 2358323 10.1016/0020-7519(90)90156-h

[15] Gardiner PR, Assoku RKG, Whitelaw DD, Murray M. 1989 Haemorrhagic lesions resulting from *Trypanosoma vivax* infection in Ayrshire cattle. Veterinary Parasitology, 31, 187–197 2763442 10.1016/0304-4017(89)90069-1

[16] Gardiner PR, Mahmoud MM. 1992 Salivarian trypanosomes causing disease in livestock outside sub-Saharan Africa, in Parasitic Protozoa, Kreier JP, Baker JR, Editors. London: Academic Press p. 277–313

[17] Hamilton PB. 2012 Is *Trypanosoma vivax* genetically diverse? Trends in Parasitology, 28, 173 22459431 10.1016/j.pt.2012.02.003

[18] Hoare CA. 1972 The Trypanosomes of Mammals. Oxford: Blackwell Scientific Publications

[19] Kabayo JP. 2002 Aiming to eliminate tsetse from Africa. Trends in Parasitology, 18, 473–475 12473355 10.1016/s1471-4922(02)02371-1

[20] Lalmanach G, Boulangé A, Serveau C, Lecaille F, Scharfstein J, Gauthier F, Authié E. 2002 Congopain from *Trypanosoma congolense*: drug target and vaccine candidate. Journal of Biological Chemistry, 383, 739–749 10.1515/BC.2002.077 12108538

[21] Magona JW, Walubengo J, Odimin JT. 2008 Acute haemorrhagic syndrome of bovine trypanosomosis in Uganda. Acta Tropica, 107, 186–191 18599006 10.1016/j.actatropica.2008.05.019

[22] Meléndez RD, Forlano M, Figueroa W. 1993 Perinatal infection with *Trypanosoma vivax* in a calf in Venezuela. Journal of Parasitology, 79, 293–294 8459344

[23] Moloo SK, Kutuza SB, Desai J. 1987 Comparative study on the infection rates of different Glossina species for East and West African *Trypanosoma vivax* stocks. Parasitology, 95, 537–542 3696778 10.1017/s0031182000057966

[24] Nakayima J, Nakao R, Alhassan A, Mahama C, Afakye K, Sugimoto C. 2012 Molecular epidemiological studies on animal trypanosomiases in Ghana. Parasites & Vectors, 5, 217 23025330 10.1186/1756-3305-5-217 PMC3480844

[25] Sajid M, McKerrow JH. 2002 Cysteine proteases of parasitic organisms. Molecular and Biochemical Parasitology, 120, 1–21 11849701 10.1016/s0166-6851(01)00438-8

[26] Shaw JJ, Lainson R. 1972 *Trypanosoma vivax* in Brazil. Annals of Tropical Medicine and Parasitology, 66, 25–32 5021570 10.1080/00034983.1972.11686794

[27] Tait A, Morrison LJ, Duffy CW, Cooper A, Turner CM, Macleod A. 2011 Trypanosome genetics: populations, phenotypes and diversity. Veterinary Parasitology, 181, 61–68 21570772 10.1016/j.vetpar.2011.04.024

[28] Vos GJ, Gardiner PR. 1990 Antigenic relatedness of stocks and clones of *Trypanosoma vivax* from East and West Africa. Parasitology, 100, 101–106 2314924 10.1017/s0031182000060169

[29] Wells EA. 1984 Animal trypanosomiasis in South America. Preventive Veterinary Medicine, 2, 31–41

